# Case report: A quantitative and qualitative diffusion tensor imaging (DTI) study in varicella zoster-related brachial plexopathy

**DOI:** 10.3389/fnimg.2022.1034241

**Published:** 2023-01-04

**Authors:** Manfredi Alberti, Federica Ginanneschi, Alessandro Rossi, Lucia Monti

**Affiliations:** ^1^Neurology Unit, Department of Neurology and Human Movement Sciences, University Hospital of Siena, Siena, Italy; ^2^Department of Medical, Surgical and Neurological Science, University of Siena, Siena, Italy; ^3^Diagnostic and Functional Neuroimaging Unit, Department of Neurology and Human Movement Sciences, University Hospital of Siena, Siena, Italy

**Keywords:** diffusion tensor imaging (DTI), varicella-zoster virus (VZV), brachial plexus, electromyography, fractional anisotropy (FA)

## Abstract

Diffusion tensor imaging (DTI) is considered feasible for the nerve plexuses' imaging and quantitative evaluation but its value in the clinical practice is still virtually unexplored. We present the DTI profile of a case of acute varicella-zoster virus (VZV)-related brachial plexopathy. A 72-year-old woman presented with left upper-limb segmental paresis involving the spinal metamers C6–C7, preceded by a painful dermatomal vesicular eruption in C5-T1 dermatomes. Clinical and electrophysiological findings and magnetic resonance imaging indicated a plexus involvement. DTI analysis showed decreased fractional anisotropy (FA) and an increase of all the other diffusivity indexes, i.e., mean, axial, and radial diffusivity. The mechanisms underlying DTI parameter differences between healthy and pathologic brachial plexus sides could be related to microstructural fiber damage. Water diffusion is affected within the nerve roots by increasing the diffusion distance, leading to increased diffusion perpendicular to the largest eigenvalue and therefore to decreased FA values The role of DTI in clinical practice has not been defined yet. Additional quantitative and qualitative DTI information could improve the assessment and follow-up of brachial plexopathy.

## Introduction

Although diffusion tensor imaging (DTI) has been widely applied to the study of the brain microstructure, its usefulness for peripheral nervous system pathology is a more recent acquisition (Hodel et al., 2011, 2022; Chhabra et al., 2013; Wade et al., 2020a,b). DTI is based on the principle that water molecules tend to diffuse more freely along the direction of axonal fascicles rather than across them. Such directional dependence of diffusivity is termed anisotropy.

DTI metrics, including fractional anisotropy (FA) and estimates of diffusivity, are sensitive to the microstructure of peripheral nerves and may provide a quantitative evaluation. In addition, DTI may offer qualitative features displayed as 3D tractography (Tagliafico et al., 2011; Chhabra et al., 2013; Gasparotti et al., 2013; Wade et al., 2020a, 2021; Fajar et al., 2022). The parameters derived from DTI are an expression of water diffusion characteristics through biological barriers and are referred to as FA, mean diffusivity (MD), axial diffusivity (AD), and radial diffusivity (RD) (Lope-Piedrafita, 2018; Fajar et al., 2022; Ranzenberger and Snyder, 2022). FA measures the degree of anisotropy of water molecules. FA depends upon the diffusion of the water molecules along a single axis (i.e., bidirectional diffusion along the length of the nerve).

AD refers to the magnitude of diffusion parallel to fiber tracts; it depends on the first eigenvalue (l1) and thus describes diffusion along the principal movement direction (Fajar et al., 2022; Ranzenberger and Snyder, 2022). AD is influenced by axonal count, caliber, and direction continuity, whereas it is not influenced by myelin integrity. Axial diffusivity is a quantification of the axonal integrity, and lower AD may reflect axonal injury, reduced axonal caliber, or less coherent axonal orientation (Ranzenberger and Snyder, 2022).

RD refers to the magnitude of diffusion perpendicular to fiber tracts and may be sensitive to myelin damage. Higher RD may reflect myelin loss or glial cell damage, reduced axonal integrity, and/or packing density. RD is the apparent water diffusion coefficient in the direction perpendicular to the axonal fibers. An increase in RD is correlated with an increased perpendicular diffusion of water molecules. This is not only correlated to tract demyelination but also to decreased axonal count and nerve degeneration due to the fact that damage to physical barriers favors an isotropic movement of the extracellular fluids (Ranzenberger and Snyder, 2022).

MD describes the average motion of water molecules, obtained from the average of the three eigenvectors (l1, l2, l3). In pathological conditions, MD should increase due to the damage of biological barriers such as myelin and cell membranes. MD is a sensitive marker of reduced white matter microstructural integrity due to either axonal or myelin damage occurring in neurological conditions such as developmental and degenerative disorders.

In the last decade, anatomical studies (Wade et al., 2020a) showed that DTI has been dedicated to pathological conditions such as traumatic, neoplastic, or inflammatory events. It has been shown that DTI may demonstrate normal tracts, tract displacement, deformation, infiltration, disruption, and disorganization of fibers due to lesions localized within or along the brachial plexus (Heckel et al., 2015; Vavasour et al., 2019; Wade et al., 2020b).

The brachial plexus has a very complex geometry which requires a 3D tractography to be analyzed. Furthermore, to differentiate brachial plexus pathologies, a 3D tractography with different angular and anisotropy thresholds for each cervical root needs to be studied (Wade et al., 2020b, 2021).

We describe the DTI profile of a case of acute varicella-zoster virus (VZV)-related brachial plexopathy.

## Case description

We report the case of a 72-year-old woman who presented with pain along the whole left upper limb. Two days later, a typical group of herpetiform vesicles on an erythematous base appeared at the level of C6 and C7 dermatomes. Within the first week, a segmental paresis of the left arm with hyperalgesia, allodynia, edema, and both color and skin-temperature changes in the left hand developed. The paresis involved the muscles innervated by C6 and C7 roots. In these muscles, there was no increase in the duration or amplitude of motor unit potentials. The lack of reinnervation of the motor units suggests that the brachial plexopathy (namely, the neurogenic muscle damage) was unequivocally recent. Anti-VZV IgM and IgG antibodies were found in the serum.

Electromyography (EMG) revealed positive sharp waves/fibrillation potentials with markedly reduced motor unit recruitment in the left extensor muscles of the wrist and the fingers, the brachioradialis, the triceps brachii, and the flexor carpi radialis. Acute denervation was absent in the muscles corresponding to the spinal metamers C5 and T1.

Based on clinical and laboratory data, a diagnosis of monoparesis of the left arm due to VZV-related brachial plexopathy was made.

### Diagnostic assessment

#### Imaging acquisition

The diffusion images were acquired on a Siemens Aera 1.5T MRI scanner using a 2D echo-planar imaging (EPI) diffusion sequence of [ep2d_diff_tensor_64_gap0], an echo time of (TE) = 81 ms, and a repetition time of (TR) = 7,100 ms. A DTI diffusion protocol was used, and a total of 64 diffusion sampling directions were acquired. The b-value was 1,000 s/mm^2^. The in-plane resolution was 2.45 mm and the slice thickness was 2.5 mm. The restricted diffusion was quantified using restricted diffusion imaging (Yeh et al., 2017). Diffusion data were reconstructed using generalized q-sampling imaging (Yeh et al., 2010) with a diffusion sampling length ratio of 1.25. The tensor metrics were calculated using DWI with a b-value lower than 1,750 s/mm^2^. A deterministic fiber tracking algorithm (Yeh et al., 2013) was used. The STIR sequence was acquired in the coronal plane (TR = 3,530 ms; TE = 56 ms; inversion time [IT] = 170 ms; flip angle = 150 degrees; slice thickness = 3 mm). MR examination was performed without intravenous administration of a contrast medium in view of the lack of sufficient information on the kidney function of the patient.

### Data sampling

An intraindividual study was performed, and the comparison was made between the healthy (right) and the pathological (left) side. The healthy side was considered as the reference standard for morphological and quantitative comparative study. The right and left sides were studied and measured with the same methodology using ROI size, symmetric (left, right) ROI position, FA range, and angular thresholds. Several FA, AD, RD, and MD measures were obtained for each ROI positioned on the nerve roots, as described in the following paragraphs.

### DTI quantitative and qualitative analyses

Quantitative and qualitative analyses were performed by using DSI studio (https://dsi-studio.labsolver.org/) on both sides (left and right) of the pathological levels, C6 and C7 roots.

To improve the diagnostic accuracy of the FA, MD, AD, and RD values, multiple measures were acquired on each involved root. Qualitative and quantitative analyses were performed using 4 different steps: (a) placing bilateral and symmetrical ROI in the proximal, medium, and distal tracts of nerve roots visualized on axial and coronal STIR images; (b) setting FA threshold values for tractogram reconstruction; c) 3D-DTI tractograms of C6 and C7 roots were obtained with different threshold values, and d) collecting the acquired diffusion parameters. Anisotropy thresholds, angular thresholds, and the minimum lengths of selected fibers were measured by considering the following values and criteria: 0.077, 0.088, 0.099, and 0.11 as anisotropy thresholds; 50, 60, and 70° as angular thresholds; and fiber tracts with lengths shorter than 15, 25, 35, and 40 mm. FA, MD, RD, and AD values were obtained along 36 different 3D-tractograms.

### Statistical analysis

The results of scaled variables are represented by the mean ± SE or SD and compared using independent samples. After verifying that the datasets fit a “not normal” distribution (Agostino-Pearson's test), DTI differences between the healthy and pathological sides were evaluated by the non-parametric Mann-Whitney's *U*-test (MWUt). Significance was set at the 5% level. The dataset was calculated on the basis of tract length series (minimum length discard criteria of 35 mm) for each anisotropy and angular threshold, respectively, 0.077, 0.088, 0.099, and 0.11 and 50, 60, and 70°. Therefore, with three series based on tract length, anisotropy, and angular thresholds, 36 measures were obtained on each nerve root for a total of 144 measurements.

## Results

We report on the quantitative analysis performed on both sides (left and right) of the pathological levels (C6 and C7 roots)

**Figure 1:**
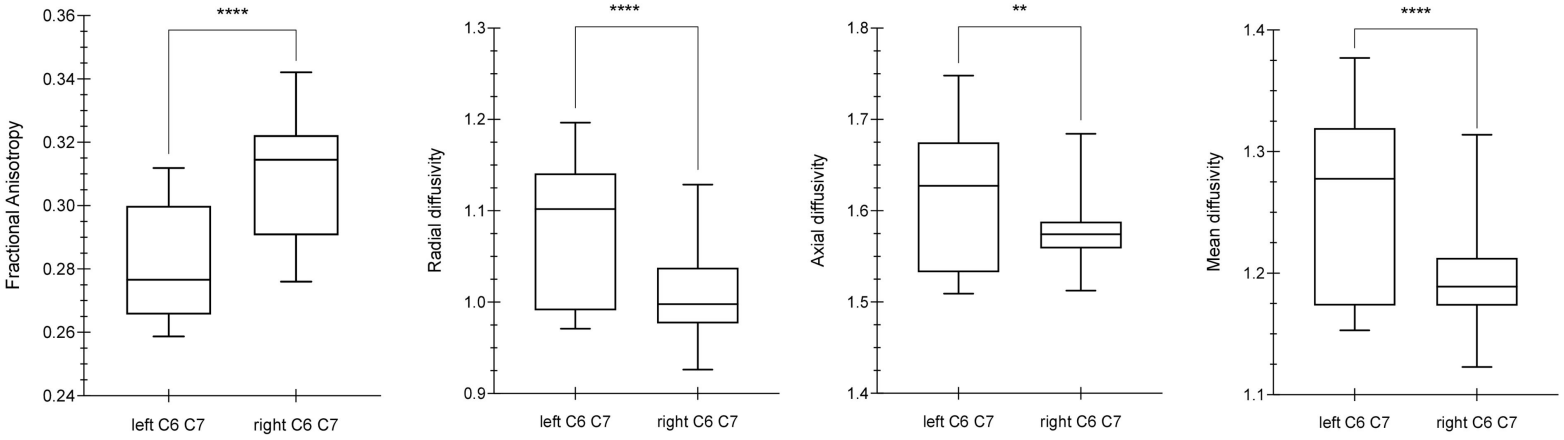
Differences between diffusion parameters in the left and right C6–C7 roots. By using the Mann Whitney U-test (MWUt), significant statistical differences were demonstrated in FA (p-value < 0.0001, MWUt = 792), RD (p-value < 0.0001, MWUt = 1,286), AD (p-value < 0.0033, MWUt = 1,861), and MD (p < 0.0001, MWUt = 1,563). The p-value lower for AD than RD suggests a minor axonal damage in comparison with dysmyelination process. **, ****indicate *p*-values significant.

Comparing the DTI values (FA, MD, AD, RD) of the C6 and C7 roots between the left and right brachial plexus, significant differences were demonstrated for FA (*p*-value < 0.0001, MWUt = 792), RD (*p*-value < 0.0001, MWUt = 1,286), AD (*p*-value < 0.0033, MWUt = 1,861), and MD (MWUt = 1,563 *p* < 0.0001) (Figure 1). Because RD and AD showed no normal distribution, a non-parametric two-tailed test was used.

Comparing the DTI values between the right and left sides for each nerve root, significant differences were demonstrated for the C6 root FA (*p*-value < 0.0001), C7 root FA (*p*-value < 0.0001), C6 root MD (*p*-value = 0.0161), C7 root MD (*p*-value < 0.0001), C6 root AD (*p*-value = 0.0423), C7 root AD (*p*-value < 0.0001), C6 root RD (*p*-value = 0.0161), and C7 root RD (*p*-value < 0.0001).

No statistically significant difference was demonstrated when assessing AD values between C6 and C7 roots of the same side. By contrast, C6 and C7 roots showed significant differences for the other DTI parameters such as FA, MD, and RD (*p*-value < 0.0001).

**Figure 2:**
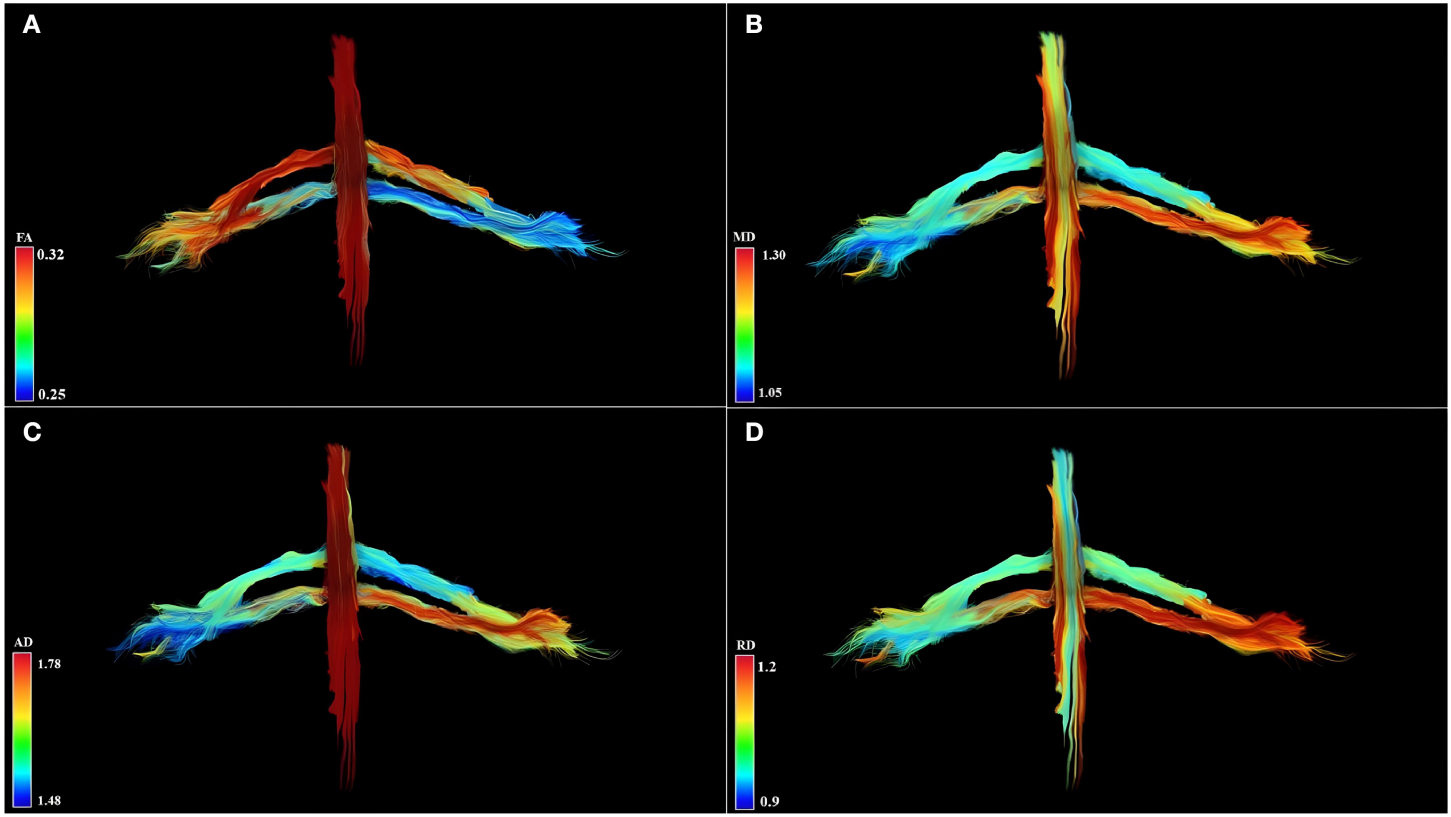
Diffusion tensor imaging tractography of the cervical cord and C6 and C7 brachial roots in three-dimensional view and parametric color maps **(A–D)**. **(A)** The color of the tract is determined by the local FA, where red denotes a high FA (0.32), scaled to yellow, green, and blue denote low FA (for rater 0.25). **(B)** The color of the tract is determined by the local MD, where red denotes a high MD (1.30), scaled to yellow, green, and blue denote low MD (for rater 1.05). **(C)** The color of the tract is determined by the local AD, where red denotes a high AD (1.78), scaled to yellow, green, and blue denote low AD (for rater 1.48). **(D)** The color of the tract is determined by the local RD, where red denotes a high RD (1.2), scaled to yellow, green, and blue denote low RD (for rater 0.9). Geometric distribution of water diffusion parameters suggests myelin damage of left C7 root, demonstrated by reduction of FA, increase of MD, AD, and RD. Left C6 root shows reduced FA values and increased MD, AD, and RD values in distal root section, suggesting a partial compromission of the nervous tissues.

Qualitative results are the 3D-DTI tractograms of the C6 and C7 roots. The C6 and C7 roots are shown with a different color score corresponding to different FA values obtaining parametric images in Figure 2. A complete background suppression was obtained.

## Discussion

In the current study, we presented the DTI characterization of a case of VZV-related brachial plexopathy, complicated by segmental paresis involving spinal metamers C6–C7.

In recent times, DTI has gained attention due to its unmatched ability to generate maps of neural pathways (qualitative approach) and provide objective proxy measures of nerve integrity (quantitative approach) (Righini et al., 2005; Wade et al., 2020a). DTI can differentiate healthy from acute and chronic neuropathy, but the complex geometry of the brachial plexus may be assessed only by considering anatomical notions such as fiber orientation within each emerging nerve, as well as data acquisition and pre- and post-processing approaches (Herweh et al., 2007; Hodel et al., 2011, 2022; Crim and Ingalls, 2017; Wade et al., 2020b; van Rosmalen et al., 2021). Indeed, the principal eigenvector of the tensor might not represent the actual fiber orientation(s) and therefore it might generate both qualitative and quantitative errors anywhere along the fiber path (Chung et al., 2013; van Rosmalen et al., 2021).

### Quantitative approach

For both central and peripheral nervous system conditions, demyelination and/or axonal injury may determine an FA decrease. For example, in chronic inflammatory demyelinating polyradiculoneuropathy and in brachial plexus injuries (Wade et al., 2020b, 2021; Su et al., 2021), the bundles of plexus nerve fibers and their branches showed lower FA. In the present case report about acute VZV-related plexopathy, a significant decrease in the FA of the brachial plexus trunks with a concomitant increase of MD, AD, and RD was demonstrated (Herweh et al., 2007; Xie et al., 2010; Meng et al., 2021; Su et al., 2021). FA decrease may be explained through knowledge of the pathophysiology of the VZV infection. In brachial VZV plexitis, virus particles infect sensory and sympathetic ganglion neurons, and considering the relationship between the ranges of impaired motor functions and the distribution of skin lesions related to this herpes virus, it is a predominant opinion that the development of segmental paresis is caused by the direct or indirect spread of VZV from the dorsal root ganglion to the spinal anterior horn cells, the anterior root, and the spinal nerves, which consequently induce motor nerve damage and functional changes (Meng et al., 2021).

Previous reports showed that patients with VZV infections have extensive lymphocytic and plasma cell infiltration in both the nerves and the ganglia (Guldberg-Moller et al., 1959; Esiri and Tomlinson, 1972). This cellular invasion is presumably prevalent in motor roots and the dorsal root ganglion, where the blood-nerve barrier is anatomically less efficient and circulating cellular and humoral immune components have easy access to these structures (Hodel et al., 2011). As a result, during the acute phase of the infection, a significant increase in edema and initial cellularity infiltration among the nerve fibers would determine an obstacle to the molecular motion, causing an anisotropy decrease (Kimura-Ohba et al., 2016). In line with this hypothesis, we observed a reduction of FA and an increase in all the other indexes of molecular displacement by diffusion. In particular, the reduced FA and the increased MD, RD, and AD values suggest the presence of fiber demyelination and axonal damage, caused by extensive axonal swelling, and an altered nerve-blood barrier due to a marked inflammatory process (Xie et al., 2010; Kimura-Ohba et al., 2016; Wade et al., 2020b, 2021; van Rosmalen et al., 2021).

The quantitative analysis performed by evaluating different FA thresholds, nerve tract lengths, and degrees of fiber direction supported the corresponding microstructural damage.

Indeed, all obtained DTI values were significantly different between healthy and pathological sides and this was consistent with clinical and electrophysiological findings. In particular, the left C7 dermatome was clinically more involved than the left C6 and these findings corresponded to DTI values, independent of post-processing thresholds. When the left C6 root (pathological side) was compared with the right C6 root (healthy side), statistical significance for the AD values was lower (p^*^) than that for the MD, RD, and FA values (p^****^) (Song et al., 2002; Wade et al., 2020b, 2021; van Rosmalen et al., 2021). AD may be related to axonal integrity. Detailed analysis of directional diffusivities (i.e., RD vs. AD) may suggest the presence of specific and prevalent pathological patterns such as demyelination and/or axonal damage. It is possible to assume that there is a smaller axonal involvement in the left C6 root than in the other pathologic root (i.e., left C7). Our findings indicate that DTI analysis has the potential to represent a pathological process in that timeframe and to detect what type of damage is prevalent.

The methodological choice to analyze different FA and degree thresholds and a selected length of fiber is crucial to reduce the errors in the quantitative approach.

In addition, our DTI analysis seems to discriminate anisotropy changes between the C6 and C7 roots on the healthy side. Indeed, FA, MD, and RD showed a significant difference when the values of the C6 and C7 roots on the right side were compared. FA, MD, and RD values of the right C7 root were higher than the right C6 root, and this could be explained by a different myelination process, although there are not exhaustive literature data in support of this hypothesis. Some authors reported that “the roots of the brachial plexus have a variable axon density, specifically 6,750 per mm^2^ in C5, 8,448 per mm^2^ in C6, 8,665 per mm^2^ in C7, 5,708 per mm^2^ in C8, and ~10,000 per mm^2^ in T1” (Won et al., 2012; Gesslbauer et al., 2017; Wade et al., 2020a, 2021; van Rosmalen et al., 2021) and these data can explain the absence of a statistical significance of the AD values between the right C6 root and the C7 root. The difference (8,448 per mm^2^ in C6 vs. 8,665 per mm^2^ in C7) is not so high as that for the other roots of the brachial plexus.

Some studies (Wade et al., 2020a, 2021) demonstrated that there are physiological differences in DTI parameters between the right and left plexus in healthy subjects, when all brachial plexus roots in all subjects, are considered. By contrast, in the single subject, no differences were demonstrated between C6 and C7 roots on both sides.

### Qualitative approach

There is a need for a 3D structure generating technique to display the anatomical characteristics of the brachial plexus. Indeed, the brachial plexus cannot be adequately represented on 2D images and standard structural MRI acquisitions are spoiled by other anatomical structures such as vascular trunks. DTI demonstrates the true geometry of the roots through 3D tractography of the brachial plexus without vascular signal contamination. The 3D-DTI tractography is the result of a multistep process, is a time-consuming procedure, and could be invalidated by the choice of type of acquisition, pre- and post-processing, due to the complexity and variants of the brachial plexus anatomy (Aung et al., 2013; Chung et al., 2013; Yeh et al., 2013; Kimura-Ohba et al., 2016; Wade et al., 2020b). Moreover, the fiber pathway reconstruction may be impaired by crossing, diverging/converging, twisting, or kinked fibers and this is a common DTI limitation for both brain and brachial plexus structural reconstruction. However, with the optimization and standardization of DTI acquisition, pre- and post-processing experts can obtain 3D modeling of the brachial plexus complex course and represent the true geometry of the roots (Figure 2).

DTI has been mostly used in experimental settings, with few indications for clinical practice. Our study may contribute to identifying brachial plexopathy through DTI.

**Figure 3:**
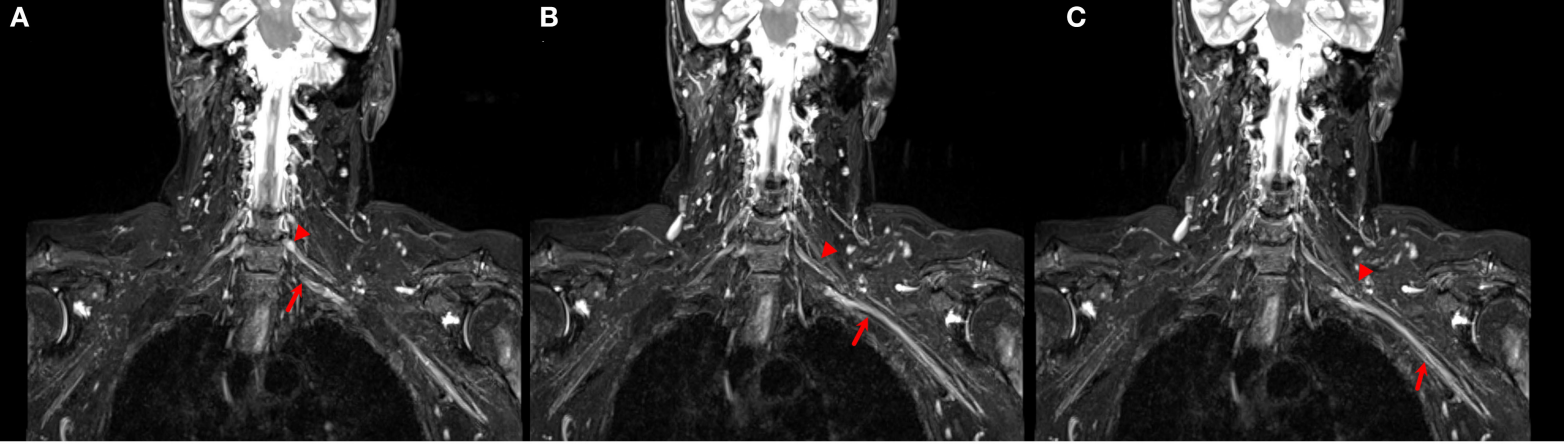
**(A–C)** Coronal STIR images of the brachial plexus. Different size and signal intensity are shown between the right and the left side of C6 (arrow head) and C7 (arrow) roots in proximal **(A, B)** and distal **(C)** tracts.

Evaluation of brachial plexus abnormalities represents a diagnostic challenge due to anatomical complexity and due to the overlapping presentation of different pathologies. Peripheral nerves and adjacent structures, for example, vessels or muscles, display similar MRI signal characteristics on STIR images (Figure 3), and differentiationbetween them may be difficult (Crim and Ingalls, 2017). Using DTI, the no-anisotropic background structures are suppressed, further improving the detection of subtle nerve abnormalities. An added value of DTI is its improved detection of morphological abnormalities of the brachial plexus (3D reconstruction) and procurement of quantitative additional information regarding the microstructure of nerve fibers, thus being a potential alternative to the use of gadolinium.

It is important to acknowledge the limitations of DTI. Motion artifacts and geometrical distortions, long acquisition time and complex post-processing procedures, the robustness of the algorithms used, and complexity of brachial plexus anatomy limit the use of DTI in clinical practice. On the other hand, DTI can be used to quantify nerve microstructural integrity, non-invasively reconstruct nerve fibers *in vivo*, and can offer valuable complementary information without vascular signal intensity artifacts and is the only advanced technology to show a 3D view of the brachial plexus. In particular, in our study, the main limitation was the complex and time-consuming post-processing procedures, while the main advantage was the quantification of the nerve damage (C6 vs. C7 roots) and its correlation with the clinical and EMG findings.

## Data availability statement

The raw data supporting the conclusions of this article will be made available by the authors, without undue reservation.

## Ethics statement

Ethical review and approval was not required for the study on human participants in accordance with the local legislation and institutional requirements. The patients/participants provided their written informed consent to participate in this study. Written informed consent was obtained from the individual(s) for the publication of any potentially identifiable images or data included in this article.

## Author contributions

LM FG, MA, and AR conceived the research, prepared the figures, drafted the manuscript, and edited and revised the manuscript. MA preprocessed the data. MA and LM analyzed the data. All authors contributed to the article and approved the submitted version.
